# Nutrient Composition, Physical Characteristics and Sensory Quality of Spinach-Enriched Wheat Bread

**DOI:** 10.3390/foods13152401

**Published:** 2024-07-29

**Authors:** Ritnesh Vishal Prasad, Sushil Dhital, Gary Williamson, Elizabeth Barber

**Affiliations:** 1 School of Chemistry, Faculty of Science, Monash University, Clayton, VIC 3800, Australia; rpra0019@student.monash.edu; 2 Department of Nutrition, Dietetics and Food, Monash University, BASE Facility, 264 Ferntree Gully Road, Notting Hill, VIC 3168, Australia; gary.williamson1@monash.edu; 3 Bioresource Processing Research Institute of Australia (BioPRIA), Department of Chemical and Biological Engineering, Faculty of Engineering, Monash University, Clayton, VIC 3800, Australia; sushil.dhital@monash.edu; 4 Victorian Heart Institute, Monash University, Victorian Heart Hospital, 631 Blackburn Road, Clayton, VIC 3168, Australia

**Keywords:** food fortification, functional food, sensory attributes, consumer acceptability, nutritional value, rheological properties, natural bioactive compounds

## Abstract

Food innovation that utilises agricultural waste while enhancing nutritional value is important for waste valorisation and consumer health. This study investigated incorporating spinach (*Spinacia oleracea*), as a model leafy agricultural waste, into wheat bread. We analysed the nutrient content, colour, texture, sensory attributes and purchase/consume intention ratings. Adding 10–40% spinach (*w*/*w*) yielded loaves with similar heights but significantly different colour and texture (*p* < 0.05) from white bread. Increasing spinach decreased total carbohydrates (including starch) while significantly increasing other nutrients (protein, fibre, iron, magnesium, potassium, zinc, calcium, vitamins A, C, E, folate, niacin, pyridoxine, nitrate/nitrite and polyphenols) (*p* < 0.05). Spinach addition increased bread porosity, linked to higher pasting parameters (peak, trough, breakdown, final and setback viscosity) with reduced pasting time and temperature. Texture analysis resulted in decreased hardness, chewiness, gumminess and firmness while increasing cohesiveness, with maximum resilience at 20% spinach enrichment. Sensory analysis with 21 untrained panellists revealed decreased visual appeal, less preferred taste, odour and overall liking (*p* < 0.05) with increasing spinach, with no significant difference in texture acceptance, but the 20% enrichment had comparable acceptance to white bread. Enriching staple foods like bread with leafy vegetable waste offers a promising approach for increasing daily vegetable intake.

## 1. Introduction

The valorisation of food waste, particularly through the incorporation of nutrient-rich components into foods to increase functionality and value, has gained significant attention in recent years. This approach not only reduces food waste significantly but also contributes to the development of functional food products to improve the health of consumers for longevity [1]. 

Inadequate consumption of fruits and vegetables is associated with poorer overall health [2]. Strong and consistent epidemiological evidence shows a lower relative and absolute disease risk with greater survival time when diet quality is improved [3,4]. Plant-based foods are reservoirs of highly bioactive health-protective compounds, among which polyphenols have shown multiple promising effects in improving overall health [5,6,7]. 

Spinach (*Spinacia oleracea*) is a green leafy vegetable with highly beneficial nutrients such as fibre, vitamins (i.e., A, C, E, K, B6 and folate) and minerals (i.e., zinc, manganese, iron, calcium, potassium and selenium) [8,9]. Raw spinach is rich in polyphenols, particularly flavonoids (i.e., luteolin, kaempferol, myricetin, quercetin and quercetagetin) [10,11,12,13]. Spinach has a global production of 30.1 million tonnes, with China leading at 27.52 million tonnes (91% of global production), followed by the United States, Turkey and Japan. Despite its high nutrient value, 35% of fresh produce is wasted during household consumption due to short shelf stability, while another ~6.5% is lost during packaging and at the retail level [14]. Given the food waste issue and inadequate fruit and vegetable intake [2], this opens an opportunity to rethink food innovation by enriching nutrient-rich ingredients, such as spinach, into commonly consumed household foods, such as bread. 

Bread is an easily accessible and digestible staple consumed by most populations. In 2023, the volume of bread production was approximately 185 billion kg, with a predicted market growth of 3.8% in 2025 [15]. Regular consumption of refined white bread low in nutrients and natural bioactive compounds can be associated with a poor diet, yet it is often more generally favoured compared to whole grain or wholemeal bread [16]. To improve the health of the population, bread could be a promising vehicle for vegetable enrichment to increase the consumption of plant bioactive compounds. We therefore assessed the physicochemical characteristics, nutrient composition and sensory properties of bread with added freshly ground spinach. We found that 20% spinach can be effectively added to the dough to produce almost equivalent sensory and physical properties to that of a control bread, with each slice of 40 g containing approximately 16 g of spinach. This manuscript should provide valuable insights and practical guidance on the integration of spinach, modelled as an agricultural waste product, into bread formulations and the assessment of sensory and physical characteristics, highlighting its potential as a newly developed food with enhanced nutritional profiles. This innovative approach not only enriches the bread with essential nutrients and bioactive compounds but also serves as a practical solution to increase vegetable consumption through a commonly consumed staple while addressing the issue of food waste.

## 2. Materials and Methods

### 2.1. Bread Formulation

Wallaby Bakers plain flour (Laucke, Strathalbyn, SA, Australia), Wallaby Bakers bread improver (Laucke, Strathalbyn, SA, Australia), instant dried yeast (Lowan Whole Foods, Glendenning, NSW, Australia), 100% gluten flour (Lotus, Kadac Pty Ltd., Braeside, VIC, Australia), salt, sugar, butter and packaged raw baby spinach (90% moisture) were purchased from a local supermarket (Coles Supermarkets Australia Pty Ltd., Hawthorn East, VIC, Australia). The basic formulation was consistent for all bread types except flour, water, gluten and blended spinach (Table 1). Spinach was freshly ground thoroughly at room temperature (25 °C) using a blender (Blixer^®®^ 2; Robot Coupe, Vincennes, France) and added to the mixture at increasing concentrations from 10% to 40% (*w*/*w*) while reducing flour by 2% (*w*/*w*) and water by 8% (*w*/*w*). Additional gluten was added to compensate for the losses of flour ratio. The control white bread consisted of 54.0% (*w*/*w*) flour and 39.9% (*w*/*w*) water without spinach. All measured ingredients were added one at a time into the dough mixer (stand mixer 4.3 L model 5KSM150. KitchenAid, Benton Harbor, MI, USA) and mixed at level 2 for ~15–20 min until the dough was no longer sticking onto the mixing bowl.

**Figure 1:**
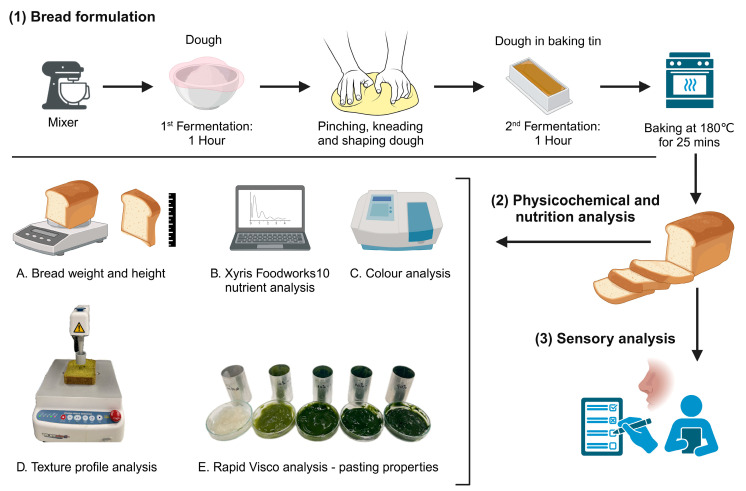
Bread formulation and subsequent analyses. (**1**) Bread formulation process, (**2**) physicochemical and nutritional analysis, (**3**) sensory analysis, *n* = 21 panellists. Created with BioRender.com. “https://app.biorender.com/illustrations/66612687f10e1aa131a6a97f (accessed on 29 June 2024)”.

The steps involved in the bread-making process used here are illustrated in Figure 1. Once kneaded thoroughly, the dough was covered and rested in a transparent bowl at room temperature (20–24 °C) for 1 h to allow the first fermentation stage. The risen dough was then punched, kneaded briefly to break large gas bubbles, shaped and placed in a bread baking tin (27 cm × 13.5 cm × 10 cm (length × width × depth)), and covered for the second fermentation stage for another hour in a temperature-controlled room of 28 °C to double in size. Once the dough was baked at 180 °C for 25 min in an oven (Rational CombiMaster® Plus CM101, Landsberg am Lech, Germany), the baked bread was left at room temperature to cool down for at least 2 h, after which the height (ends and mid-points) and weight were recorded. This process was repeated in triplicate for each bread for physicochemical and nutrient analysis. The crust of the freshly baked bread samples was removed before sensory analysis.

### 2.2. Physiochemical and Nutritional Analysis

#### 2.2.1. Bread Height 

The height of the bread was measured at the mid-point using a ruler in triplicate obtained from bread samples baked on different days. 

#### 2.2.2. Nutrient Composition Analysis 

The nutrient composition of the bread was calculated based on its ingredients’ composition using FoodWorks10 Professional software (Xyris Pty Ltd., Spring Hill, QLD, Australia). The weight of ingredients, total yield and weights before and after baking were entered into the software, where the yield was calculated using the following formula:

(1) $$\mathrm{Yield}\;(\%)=\frac{\mathrm{final}\;\mathrm{baked}\;\mathrm{weight}\;(g)}{\mathrm{raw}\;\mathrm{dough}\;\mathrm{weight}\;(g)}\;\times \;100\%$$

Retention factors were applied to spinach and flour to account for the nutrient losses during the baking process. The nitrate (296 mg/100 g) and nitrite (3.8 mg/100 g) content adapted from [17] and the total polyphenol (248.14 mg/100 g), kaempferol (7.86 mg/100 g), quercetin (5.87 mg/100 g) and luteolin (1.11 mg/100 g) content adapted from [11] were manually added to the FoodWorks10 Professional software for data analysis.

#### 2.2.3. Colour Analysis

The colour of baked bread was analysed using ColorFlex EZ (HunterLab, Reston, VA, USA). Samples (25 mm) were placed in HunterLab sample cups and analysed in triplicate. Values of L*, a*, b* and ΔE* were obtained as previously described [18].

#### 2.2.4. Texture Profile Analysis (TPA)

The texture profile of the bread was analysed using a TA.XT Plus texture analyser (Stable Micro Systems, Godalming, England, UK). TPA was performed in accordance with the American Association of Cereals Chemists (AACC) Standard 74-09 [19], specified by the Exponent Connect Software, as reported previously [20]. The crust was removed, and the bread was sliced into equal thicknesses of 25 mm using a ruler and a knife. A 36 mm radius cylindrical flat probe with a 5 kg load cell was used for the double compression test at a 50% compression rate, with a test speed of 1.7 mm/s and a post-test speed of 10 mm/s performed in duplicate. While the firmness (g) and hardness (g) values were provided by the software, the remaining parameters were calculated as follows:

(2) $$\mathrm{Springiness}\;(\%)=\frac{\mathrm{distance}\;\mathrm{of}\;2\mathrm{nd}\;\mathrm{compression}\;(\mathrm{mm})}{\mathrm{distance}\;\mathrm{of}\;1\mathrm{st}\;\mathrm{compression}\;(\mathrm{mm})}\;\times \;100\%$$

(3) $$\mathrm{Cohesiveness}\;(\%)=\frac{\;\mathrm{area}\;\mathrm{under}\;\mathrm{the}\;2\mathrm{nd}\;\mathrm{compression}\;\mathrm{curve}\;(g.s)}{\mathrm{area}\;\mathrm{under}\;\mathrm{the}\;1\mathrm{st}\;\mathrm{compression}\;\mathrm{curve}\;(g.s)}\;\times \;100\%$$

(4) $$\mathrm{Resilience}\;(\%)=\frac{\;\mathrm{area}\;\mathrm{under}\;1\mathrm{st}\;\mathrm{compression}\;\mathrm{curve}\;\mathrm{during}\;\mathrm{recovery}\;\mathrm{phase}\;(g.s)}{\mathrm{area}\;\mathrm{under}\;1\mathrm{st}\;\mathrm{compression}\;\mathrm{curve}\;\mathrm{during}\;\mathrm{compression}\;\mathrm{phase}\;(g.s)}\;\times \;100\%$$

(5) Chewiness (g)=hardness (g) × cohesiveness × springiness

(6) Gumminess (g)=hardness (g) × cohesiveness

#### 2.2.5. Pasting Properties

The spinach was first freeze-dried (Labconco Corporation, Kansas City, MO, USA) and was then ground using a mortar and pestle before sieving through a 300 µm sieve to obtain a homogenised and consistent powder. The powder and the flour samples were placed in a drying oven (N-BIOTEK, Inc., Gyeonggi-do, Korea) overnight at 40 °C to equalise moisture content before measuring the pasting characteristics. 

The pasting properties of dough were analysed using a Rapid Visco Analyser (RVA) 4500 (Perten Instruments, Stockholm, Sweden). Flour (3.0 g dry basis) and spinach powder of 10%, 20%, 30% and 40% (*w*/*w*) dry weight were added to a canister and topped up with distilled water to obtain a constant weight of 28 g flour–spinach mixture. The slurry was homogenised using an RVA spindle. For RVA measurements, the samples underwent a temperature cycle: 50 °C for 1 min, increased to 95 °C for 3.75 min, held at 95 °C for 2.5 min, cooled to 50 °C for 3.75 min and then maintained at 50 °C for 2 min. After the analysis, peak, trough, breakdown, final viscosity, setback, peak time and the pasting temperature were obtained to determine the pasting profiles, as adapted from [21,22].

### 2.3. Sensory Evaluation and Food Purchasing Ominr Consumption Rating Test

**Figure 2:**
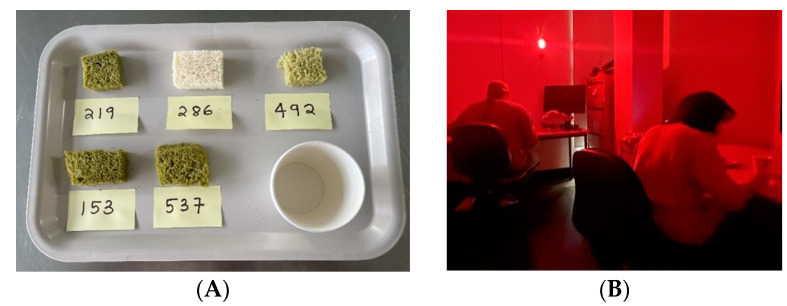
Sensory evaluation of bread. (**A**) Samples presented on a tray with three-digit codes with water. (**B**) Room with red lighting used to conduct sensory evaluation.

The sensory evaluation was performed following the ISO 6658:2017 standard [23] using 21 untrained panellists over 18 years old, obtained from the Department of Nutrition, Dietetics and Food, Monash University. The ethics was approved by the Monash University Human Research Ethics Committee (MUHREC #26497). A minimum of 20 panellists was sufficient to provide adequate heterogeneity in consumer preferences, as previously mentioned [24]. The bread crust was removed, and the crumb was cut into bite-size pieces, packed and refrigerated (4 °C) for no more than 2 days for sensory evaluation. The samples were thawed to room temperature for >30 min before serving to the panellists for assessment, in randomised three-digit codes (Figure 2A) to conceal the identities. Sensory analysis was conducted in a room with red lighting (Figure 2B) to mask the colour and appearance of the green hue to minimise bias, modified from [25]. The 9-scale hedonic rating test and 7-scale purchase or consume intent rating tests were used for sensory evaluation. The hedonic scale test consists of 1 as “dislike extremely”, 5 as “neither like nor dislike” and 9 as “like extremely”. The sensory evaluation was conducted at 0930-1130 (between breakfast and lunch) when the panellists were not too hungry or full. During the evaluation, the panellists tasted one sample at a time, rinsed mouths in between with water, and evaluated taste, appearance, odour, texture and overall liking attributes as described previously [23]. 

The 7-point purchase or consume intent rating test was presented with a set of statements as follows:

Score 7—“I would buy/consume this product at every opportunity I had”
Score 6—“I would buy/consume this product very often”
Score 5—“I like this product and would buy/consume it now and then”
Score 4—“I would buy/consume this product if available but would not go out of my way”
Score 3—“I do not like this product but would buy/consume it on occasion”
Score 2—“I would hardly ever buy/consume this product”
Score 1—“I would buy/consume this product only if forced to”

Greater scores (5–7) indicated high purchasing/consumption intention, a score of 4 indicated neutrality and lower scores (3–1) indicated negative purchasing or consumption perception. The original method [26] was slightly modified by having numerical scores (1 to 7) to facilitate data analysis. The terms “buy” and “consume” were used to capture consumers’ purchasing and consumption intentions, respectively.

### 2.4. Statistical Analysis and Illustrations

The data were analysed using one-way analysis of variance (ANOVA) to compare the different means. Tukey’s test was selected for multiple comparisons with statistical significance at *p* < 0.05. GraphPad Prism (version 10.0.2, San Diego, CA, USA) software was used to conduct statistical analysis and prepare graphs. The nutrient composition of the bread was assessed using Foodworks10 Professional software (Xyris Pty Ltd., Spring Hill, QLD, Australia). Figure illustrations were created using the BioRender.com application. 

## 3. Results

### 3.1. Bread Formulation

**Figure 3:**
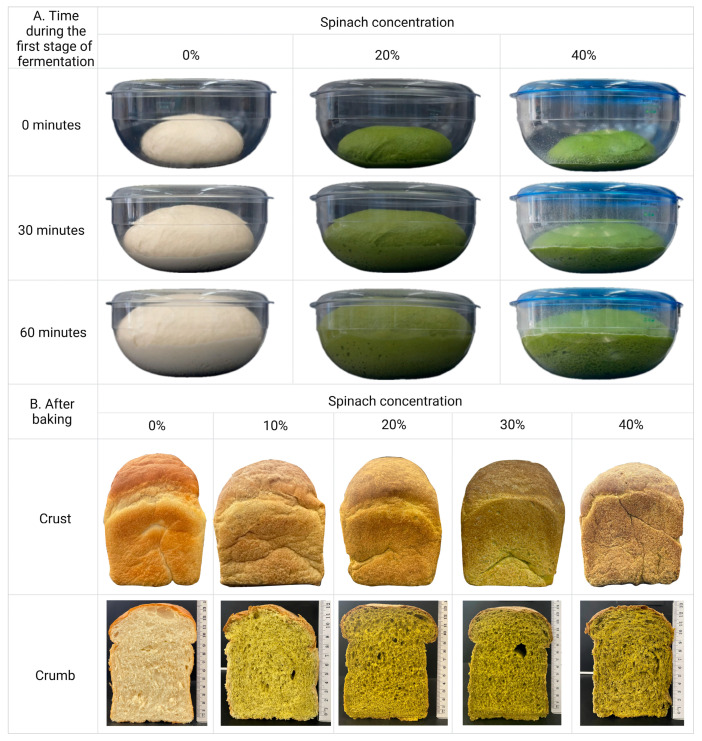
Dough expansion and bread characteristics. (**A**) Dough fermented for 0, 30 and 60 min with 0%, 20% and 40% spinach enrichment. (**B**) Bread crumb and crust characteristics for 0%, 10%, 20%, 30% and 40% spinach-enriched bread. Created with Biorender.com. “https://app.biorender.com/illustrations/66a1a1c7fddd4fb6c3081b2e (accessed on 29 June 2024)”.

Increasing the spinach concentration in the dough affected the colour, texture and fermentation behaviour of the bread (Figure 3).

The dough with 40% spinach had an apparent wetter texture during fermentation than the control, causing some difficulty during the handling and kneading processes. As the spinach concentration increased, there was a noticeable decrease in dough expansion upon fermentation, while an increased green hue in the crumb and crust colour in the baked products was noted (Figure 3).

**Figure 4:**
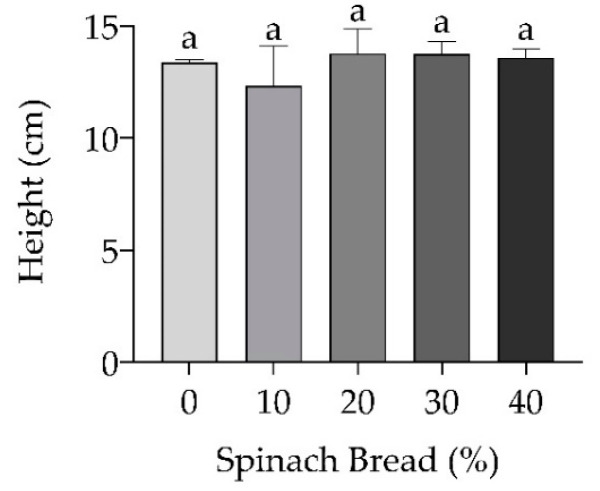
Bread height with and without spinach enrichment. Different letters indicate significant differences at *p* < 0.05. Values are presented as mean ± SD, *n* = 3.

Table 2 indicates the decrease in yield as the spinach concentration increased. The highest yield was observed in control white bread (84%), followed by 10%, 20% and 30% spinach bread (*p* > 0.05), while the lowest yield was shown in 40% spinach bread (*p* < 0.05). Similarly, the weight was highest in white bread (749 g), followed by 10% and 20% spinach bread (*p* > 0.05), while the 30% and 40% spinach bread had a 4.8% reduction in weight (*p* < 0.05). Despite the reduced dough expansion observed during fermentation, bread height was unaffected by increasing spinach concentration (Figure 4). Spinach enrichment seemed to lower the yields and weights by almost 5% without affecting the height of the baked products.

### 3.2. Nutrient Composition

The nutritional composition of the samples was determined using FoodWorks10 Professional software, which reflects nutrient data sourced from AusFoods, AusBrands, FOODfiles (NZ) and the USDA National Nutrient Database. AusFoods includes nutrient data from AUSNUT 2011-13 and the most current Australian Food Composition Database (AFCD), whereas AusBrands provides data collected from nutrition information panels of commercial products in Australia, linked to AusFoods for a more comprehensive nutrient profile [27,28].

Nutrient analysis of spinach enrichment in bread revealed significant improvements in several key nutritional parameters, including macronutrients, micronutrients and bioactive compounds, compared to white bread (Table 3 and Table 4). With increasing spinach concentration, the energy and polyunsaturated fat content decreased by 5% (*p* = 0.016) and 12% (*p* < 0.001), respectively, in the highest spinach enrichment compared to the control, without affecting the total fats, saturated fats and trans fatty acid content throughout. As predicted, the replacement of gluten content in spinach-enriched bread (due to the lesser gluten-fortified bread flour used) increased the protein content by 14% in the highest spinach-enriched bread (*p* = 0.0002). 

Total carbohydrate content decreased by 7% (*p* = 0.012) and 10% (*p* = 0.0009) in the 30% and 40% spinach-enriched bread, respectively. This is evident by a significant 12% (*p* = 0.0007) decrease in starch content, although a slight increase in sugar in 20%, 30% and 40% spinach bread were noted (*p* < 0.05). Dietary fibre and ash content increased significantly by 53% and 70%, respectively (*p* < 0.0001). The nutrient analysis (Table 3) suggests enhanced nutritional values, with a significant increase in protein, fibre and ash content while decreasing total carbohydrates, especially starch. 

Thiamin content remained relatively stable across all samples, indicating that spinach incorporation did not affect this vitamin. Spinach is a good source of riboflavin and niacin, so its addition proportionately increases these nutrients in bread. The pyridoxine and folate levels increased significantly, maximising by a 140% and 116% increase, respectively, as the spinach enrichment reached 40% (*p* < 0.05). Composite antioxidant vitamins A, E and C increased significantly with increasing spinach concentration. Vitamin A and E increased by nearly 15- and 8-fold, respectively (*p* < 0.05), while vitamin C increased from 0 mg to 9.44 mg at the highest spinach concentration (*p* < 0.0001).

Mineral content significantly increased with increasing spinach concentration in bread, where iron increased by ~threefold, while calcium, potassium and magnesium nearly doubled (*p* < 0.0001). At the highest spinach enrichment, zinc and phosphorus elevated to 33% and 17%, respectively (*p* < 0.05). The sodium content was similar between the control and lower spinach enrichment and only increased to 9% in the highest spinach enrichment (*p* < 0.05). Despite the increase in mineral content, selenium decreased significantly by 5% (*p* = 0.013) in the 40% spinach bread but no difference occurred in the others.

Nitrites and nitrates were significantly increased in all spinach-enriched bread formulations (*p* < 0.0001). The total polyphenols, including kaempferol, quercetin and luteolin levels exhibited pronounced increases from 0 mg/100 g in white bread to a maximum of 123.3 mg/100 g, 3.93 mg/100 g, 2.93 mg/100 g and 0.55 mg/100 g in 40% spinach-enriched bread, respectively (*p* < 0.0001). The enhancement in the nutrient content of the spinach-enriched bread is primarily attributed to the spinach enrichment (Table 3 and Table 4), indicating that the addition of spinach significantly boosts the overall nutritional profile.

### 3.3. Pasting Properties

**Figure 5:**
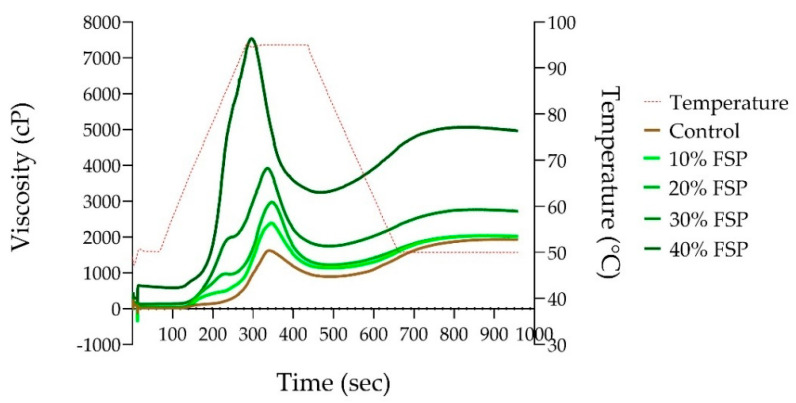
Pasting properties of wheat flour with and without enrichment with 10%, 20%, 30% and 40% freeze-dried spinach powder (FSP). Values are the mean of duplicate samples.

Pasting properties are measured using viscosity parameters during the heating and cooling of starch to help understand the texture, quality and stability of the final product. In this study, freeze-dried spinach powder was added at 10%, 20%, 30% and 40% and compared with the control during viscosity measurement (Figure 5 and Table 5). The peak, trough, breakdown, final and setback viscosity increased significantly (*p* < 0.05) while the peak time and pasting temperature decreased with increasing spinach concentration from 10% to 40% compared with the control (Table 5).

Peak viscosity, maximum viscosity achieved through the hydration, swelling and disruption of starch granules during heating [31,32,33] increased gradually with increasing concentrations of spinach powder and reached almost fivefold at 40% (*p* < 0.05). The gradual increase in peak viscosity with an increase in spinach from 10% to 40% suggests the additive and synergistic effects due to swelling of spinach fibres. The stability of the paste became stronger and followed the trend of peak viscosity, gradually increasing with increasing concentrations of spinach powder, maximising to 3.6-fold at 40% (*p* < 0.05). Trough viscosity (or hot paste viscosity) is the minimum viscosity reached after the peak during the cooling phase, representing the stability of the paste to shear [34,35,36]. With increasing concentrations of spinach powder, breakdown viscosity (peak-trough) gradually increased to almost sixfold (*p* < 0.05). It is noted that the ratio of peak to trough is increased with an increase in spinach powder, suggesting resistance to the breakdown of the paste during the holding and cooling phases in RVA.

Final viscosity reflects the ability of starch to form a viscous gel or paste upon cooling, determined as soluble amylose retrogradation obtained at the end of the cooling phase at 50 °C [33,35,36]. The final viscosity increased gradually with increasing spinach concentration (*p* > 0.05), maximised to 2.6-fold at 40% (*p* < 0.05). Setback viscosity reflects the reassociation of amylose and amylopectin into an ordered structure when starch molecules undergo retrogradation, promoting starch recrystallisation. This is determined by the difference between the final viscosity and trough viscosity [33,35,36]. The setback viscosity did not change with an addition ranging from 10% to 30% spinach powder (*p* > 0.05) but increased significantly in 40% (*p* < 0.05) compared with the control, indicating increased retrogradation and syneresis. It should be noted that in a complex system, i.e., the addition of spinach powder, the final viscosity as well as setback not only affects the function of starch pasting but also is affected by water absorption and retention by additional non-starch components. 

Peak time is the time taken to reach peak viscosity, reflecting the rate at which starch granules hydrate, swell and rupture [33,36]. The peak time was similar to the control when 10% and 20% spinach powder was added but decreased significantly at 30% and 40% (*p* < 0.05), indicating that spinach addition accelerates gelatinisation. The pasting temperature determines the point at which viscosity begins to rise (onset of starch gelatinisation), indicating the minimum temperature required to cook a product [33,36]. The pasting temperature decreased significantly (*p* < 0.05) with the addition of spinach powder, indicating that spinach addition interfered with starch swelling, most likely due to providing a physical barrier (e.g., encapsulating or covering the starch) or by limiting the availability of water. It should be noted that spinach powder has a higher water absorption capacity than wheat starch. 

### 3.4. Texture Profile Analysis

**Figure 6:**
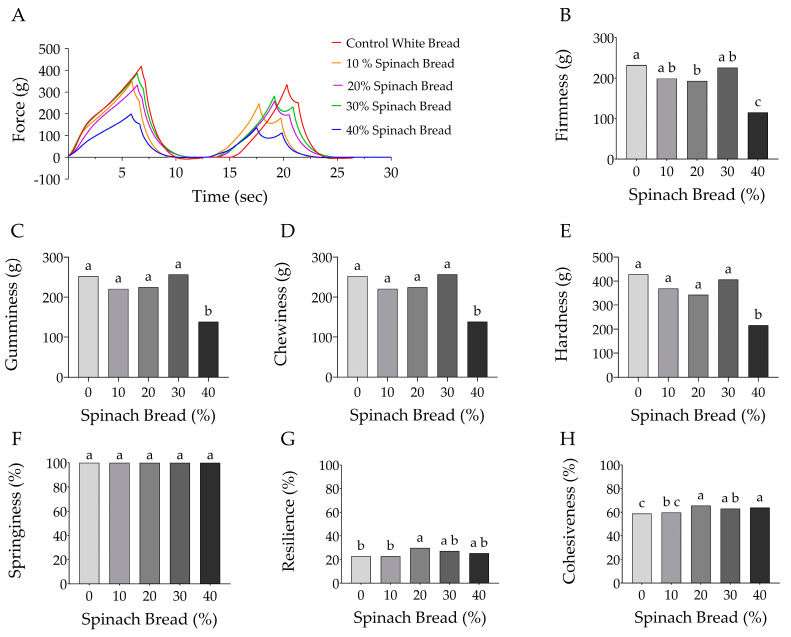
(**A**) Texture profile analysis and texture analysis parameters (**B**) firmness (g), (**C**) gumminess (g), (**D**) chewiness (g), (**E**) hardness (g), (**F**) springiness (%), (**G**) resilience (%), (**H**) cohesiveness (%) of control white bread and spinach-enriched bread at various concentrations from 10% to 40% (*w*/*w*). Different letters indicate significant differences at *p* < 0.05. Values are the mean of duplicate samples.

Texture profile analysis employs a double-mechanical compression test which helps understand the chewing behaviour of food. Firmness, hardness, springiness, cohesiveness, chewiness, gumminess and resilience are common parameters that make up the texture profile of food products such as bread. Significant variations were observed in the texture profiles of the control and spinach-containing breads (Figure 6). 

Firmness is determined by the amount of force required to deform or rupture a food sample [37], indicating a soft texture. Gumminess is used to describe the energy needed to break down a semi-solid food before swallowing, which is calculated by multiplying the hardness and cohesiveness of the food [38]. Chewiness is determined by the product of hardness, cohesiveness and springiness, which together represent the level of effort required to chew food to a consistency that is ideal for swallowing. [39,40]. Crumb hardness is measured by the maximum force during the first compression [41], reflecting the compression force between the molars (using incisors to bite through) and the compression force between the tongue and palate [39,42]. 

The control bread had the highest firmness, gumminess, chewiness and hardness, which decreased gradually with the addition of spinach, and the lowest at 40% (*p* < 0.05), reflecting the positive influence of spinach on bread softness and chewability. 

Springiness and resilience are reverse measures when breadcrumbs are compressed by a tool. Springiness was similar in all samples with and without spinach (*p* < 0.05), indicating that spinach addition did not affect the deformation of bread crumb shapes when compressed. This means that the addition of spinach to bread did not affect its viscoelastic properties. Resilience determines elasticity, measured by the ability of a food to quickly return to its original height after being compressed [33]. Maximum resilience (30%) was shown by 20% spinach bread, which was significantly lower in the control bread and 10% spinach bread, but not different to the 30% and 40% spinach bread (*p* > 0.05). This indicates that moderate to high spinach enrichment improves elasticity.

Cohesiveness is a measure of how well the product withstands a second deformation relative to the first deformation [39], which is somewhat affected by resilience. The 20% spinach bread showed the highest cohesiveness, similarly, shown by 30% and 40% spinach bread, but significantly lowered in the control white bread and 10% spinach bread (*p* < 0.05). This indicates a stronger ability to hold the internal structure together at 20% spinach concentration.

### 3.5. Colour Analysis

**Figure 7:**
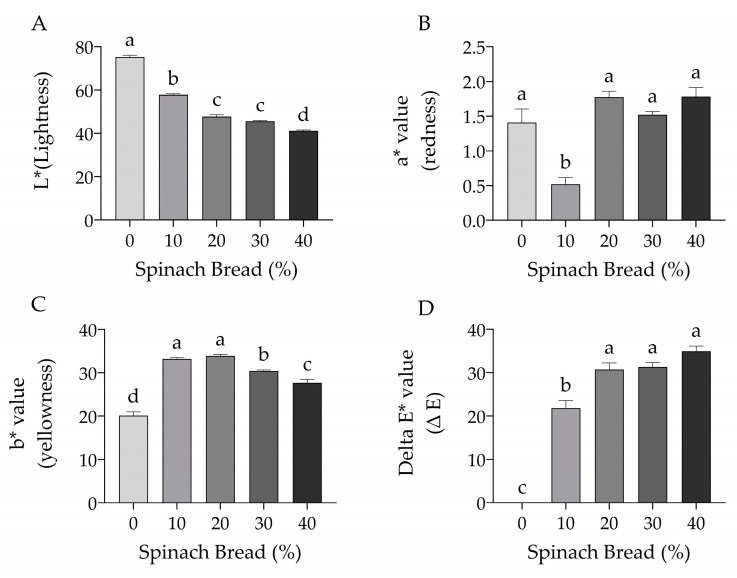
The colour analysis of bread with and without the addition of spinach at 10%, 20%, 30% and 40% (*w*/*w*). (**A**) L* value: lightness/brightness (0: black to 100:white), (**B**) a* value: redness (+a*) or greenness (−a*), (**C**) b* value: yellowness (+b*) or blueness (−b*) and (**D**) ΔE* value: overall colour difference. Different letters indicate significant differences at *p* < 0.05. Values are mean ± SEM, *n* = 3.

Colour often influences consumers’ acceptance and liking of certain foods. In spinach, the presence of naturally coloured compounds (e.g., yellow, red and orange carotenoids) is often masked by abundant green chlorophyll [43]. In this study, colour parameters were measured using the values of lightness (L*), redness (a*), yellowness (b*) and overall colour difference (ΔE*) to assess their correlations with consumer perception towards the bread varieties. Colour analysis reveals significant changes in colour parameters with the addition of spinach (10%, 20%, 30% and 40%) compared to the control white bread (Figure 7). 

Increasing spinach content led to a significant decrease in the lightness (L*) of the bread (*p* < 0.05) (Figure 7A). White bread showed the brightest colour, which gradually decreased with increasing spinach concentration. The highest spinach concentration (40%) resulted in the darkest bread (also reflected in Figure 3). Alternatively, redness (a*) showed no difference with or without the addition of spinach except for 10% spinach bread (*p* < 0.05) (Figure 7B). The decrease in redness at 10% spinach enrichment may be due to the dilution effect of carotenoids, resulting in a more prominent green colour from chlorophyll. The yellowness (b*) was highest at the 10% and 20% spinach bread, gradually decreasing with increasing concentration of spinach at 30% and 40% and lowest in the control white bread (*p* < 0.05) (Figure 7C). The overall colour difference (ΔE*) increased significantly with increasing concentrations of spinach compared to white bread (*p* < 0.05) (Figure 7D). 

### 3.6. Sensory Analysis

**Figure 8:**
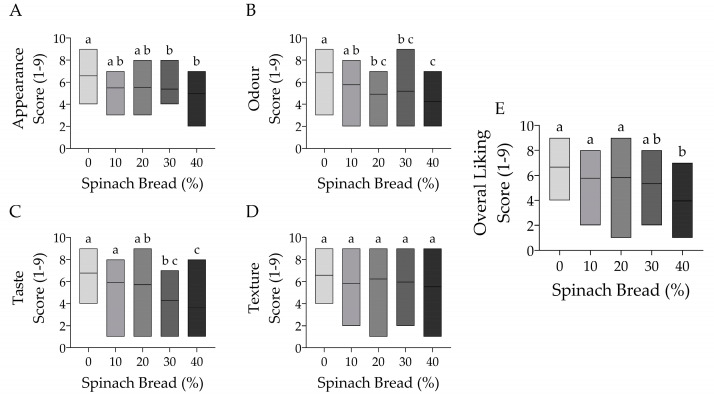
Sensory analysis of control white bread compared with spinach-enriched bread at varying concentrations of 10–40% (*w*/*w*), assessing (**A**) appearance, (**B**) odour, (**C**) taste, (**D**) texture and (**E**) overall liking between scores 1–9. Different letters indicate significant differences at *p* < 0.05. The middle line in the bar graph represents the mean (*n* = 21).

The sensory analysis of the breads was analysed in two ways: a 9-point hedonic scale test to measure their levels of preference for certain attributes, and a 7-point consumers’ future purchasing or consuming intention test. The hedonic scale test reveals the consumer preferences for bread enriched with and without spinach (Figure 8). Despite the analysis conducted in a red-lighting room presented as coded samples to conceal the colour, the highest liking to appearance, odour and taste was demonstrated by the white bread with no differences in texture acceptance between groups (*p* > 0.05). The bread with 10% spinach showed similar acceptance in appearance, odour and taste, while the 20% spinach addition had similar appearance and taste acceptance to the control bread (*p* > 0.05). The overall liking only decreased with the addition of 40% spinach (*p* < 0.05).

Despite a positive hedonic rating for the spinach-enriched bread, particularly at 10% and 20%, the panellists provided several comments and recommendations to improve the spinach-enriched bread, revealing various consumer perceptions for future product improvement (Table 6). White bread was the most preferred option based on positive feedback, while the spinach-enriched bread received criticism for its odour and bitterness. The negative comments intensified with higher spinach concentrations, with 40% being the most disliked, having the most recommendations, due to the highest degree of bitterness, unpleasant aftertaste and odour. Surprisingly, participants commented on the dry texture of the control white bread and 10% spinach bread but not on the 20%, 30% and 40% spinach-enriched bread. The lack of saltiness is commented on throughout; however, sourness and bitterness are more pronounced in the spinach-enriched bread, coupled with a strongly disliked odour. 

**Figure 9:**
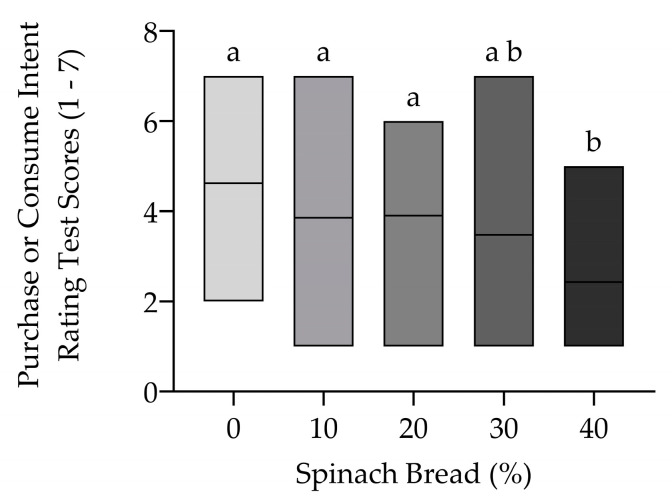
Purchase or consume rating test of control white bread compared to spinach-enriched bread at varying concentrations from 10% to 40% (*w*/*w*) between scores 1–7. Different letters indicate significant differences at *p* < 0.05. The middle line in the bar graph represents the mean (*n* = 21).

The consumer’s future purchasing or consumption intention test uses a scale of 1 to 7, scoring from the lowest (1) to the highest (7) frequency of willingness to purchase or consume the product. A high mean score (4.62) is received by the control white bread that is not significantly different from spinach-enriched bread at 10% and 20%, scoring 3.86 and 3.90, respectively, with a slight decrease at 30% (*p* > 0.05) (Figure 9). This means that moderate spinach enrichment (20–30%) is well accepted and participants would still purchase or consume the bread frequently. A significant reduction in purchasing or consuming score is most obvious in 40% spinach-enriched bread (*p* < 0.05) and is consistent with the most disliked type observed from the hedonic rating test. Disliking certain characteristics of the bread influences the frequency of consumers’ intention to purchase or consume in the future. 

## 4. Discussion

Waste valorisation has gained significant attention in the food industry due to its dual benefits of reusing sustainable ingredients and enhancing the nutritional profile of food products. Incorporating spinach, a nutrient-rich green leafy vegetable, into staple bread is one example of waste valorisation. Several plant ingredients, such as banana, mung bean and moringa leaves, have been used earlier to increase the nutrient composition of bread [44,45,46]. Our study indicates that spinach-enriched bread exhibits improved nutritional qualities, with acceptable physical and sensory attributes, suggesting potential for innovative and sustainable food products. 

Achieving the best formulation for the bread dough was challenging due to the water present in the raw spinach that interfered with the initial product development process. Although several others have formulated bread using freeze-dried vegetables [47,48], in our study, fresh spinach was used to minimise the losses of processing-sensitive nutrients, particularly polyphenols. In addition, the use of frozen spinach developed an unpleasant green hue on the crust and crumb (unpublished results) and was, therefore, not pursued further. 

Dough with the highest amount of added spinach showed reduced expansion (5%) upon fermentation due to high fibre content. It is known that fibre-rich doughs have high water absorbability and reduced fermentation tolerance, resulting in shorter doughs [49]. Fibre disrupts the gluten network by interfering and competing with starch and gluten for hydration, resulting in compromised strength and cohesiveness of the dough shown by greater stickiness during handling and kneading processes [50]. Hence, with increasing fibre content in spinach, more prominently in 40% enrichment, greater stickiness reduced the total weight of dough due to losses on the surface, which contributes to lesser bread yield when baked. Moreover, the fibre in spinach enhanced the pasting properties of starch, evident from the increased viscosity parameters. The fibre in the starch matrix resulted in increased viscosity, porosity and stability, reflected by a softer texture. This may be due to the high water absorption and holding ability of fibres compared to starch. Further, the physical degradation (shear scission) of fibres is comparatively lower than that of starch polymer, leading to the increase in trough and final viscosity proportionate to the addition of spinach powder. Additionally, the high water absorption, water-holding capacity and the ability to form a networked gel structure of these fibres [33,51] further contributed to the softness of baked bread without being dry. Similar pasting behaviour was also observed with other high-fibre ingredients, such as oats, peas, lemons, apples [33] and kiwi [51]. Along with fibres, the polyphenols in vegetables interact with the starch matrix to increase viscosity in the starch system [52]. The increased gelatinisation characteristics could be due to the expansion of amylopectin and the molecular dissolution of amylose in the starch-polyphenol system at high temperatures [21]. This means that both the fibre and polyphenols in spinach can potentially influence the gelatinisation process, affecting the pasting properties of bread, and altering the sensory properties of the final products. 

The resulting baked bread had similar physical characteristics with or without spinach enrichment owing to the extra added gluten, unlike previous reports, which did not have extra gluten [44,45,46]. When wheat flour is mixed with water, gluten proteins help create a cohesive dough that traps gases during proofing, essential for producing desirable structural characteristics of soft-leaven bread when heated. Without gluten, doughs lack cohesion and elasticity, affecting the final rheological properties of the bread [53]. In this study, we propose that the interaction between gluten proteins and spinach fibre improved the final texture through noncovalent bonds [54,55,56,57], resulting in a denser crumb porosity and better water-holding capacity and firmness with reduced chewiness. 

Naturally coloured bioactive compounds such as green chlorophyll and red–orange–yellow carotenoids are prominent in spinach leaves [43], and so the amount of spinach added to the bread affected the colour of the final crumb and crust of the bread. The decrease in lightness values in the spinach bread can be attributed to the degradation or conversion of green chlorophylls (chlorophyll a and b) into olive-brown products (such as pheophytins, pheophorbides, pyropheophytins and pyropheophorbides) during baking [43,58]. The redness at a lower spinach enrichment level is masked by a high chlorophyll content, showing a significant reduction in 10% spinach bread, while higher stability of carotenoids compared to chlorophylls was observed during heat treatment, as reported earlier [43]. Similar results were not seen when powdered vegetables were used [18,57,59], demonstrating losses in bioactive compounds when fresh ingredients are not used. In contrast, the decrease in yellowness is less affected by green chlorophyll, probably due to their higher abundance than the thermal-sensitive red pigments [43] or produced from the Maillard reactive products following the interactions between amino acids and reducing sugars, similarly found earlier in spinach-enriched flatbread [60]. The green hue was most preferred at 10 or 20% spinach concentrations, while further increases were disliked in appearance and overall liking, consistent with previous studies when spinach and moringa powders were introduced into bread [18,57,60,61]. 

In general, spinach enrichment affected the taste and odour of the final products. While spinach at lower levels (10% or 20%) did not significantly affect the sensory attributes and purchasing or consumption rating of the bread, higher concentrations (30% or 40%) resulted in lesser acceptance, consistent with several other reports [18,44,57,60]. Bitterness is apparent with increasing spinach concentration, confirming the astringency of spinach polyphenols [44]. Polyphenols from spinach have been shown to interact with the taste receptors T2R39 and T2R60 [62], and the strong grassy and sulphurous odour of spinach is partly due to dimethyl sulphide, methanethiol and dimethyl trisulphide [63,64,65]. Nevertheless, spinach enrichment at 20% demonstrated the highest acceptance of most sensory characteristics, with few differences from white bread. 

The incorporation of spinach in the bread enhanced its nutritional value. While the reduced starch and carbohydrate levels resulted from the reduced flour amount in the formulation, the slight increase in protein and sugar in spinach-enriched bread reflected following the protein and sugar present in spinach at 2.4 g/100 g FW and 0.6 g/100 g FW [29]. Fresh spinach is rich in the antioxidant vitamins A (469 µg/100 g FW) [30], C (28.1 mg/100 g FW) and E (1.3 mg/100 g FW) [29], as well as nitrates, nitrates and polyphenols. Hence, these vitamins become substantially increased upon enrichment, supporting a potential health benefit [66]. Previous incorporation of vegetables into bread [48,67,68,69] consistently showed increased protein, fibre, phenolic compounds and several micronutrients. While there may be some antagonistic effects due to the presence of compounds like phytic acid and oxalates [70], the overall nutritional benefits of spinach-enriched bread outweigh these effects. The synergistic effects, particularly the enhanced micronutrient, fibre and polyphenol content make spinach-enriched bread a nutritionally superior option compared to regular white bread.

While WHO recommends a daily intake of 400 g of fruits and vegetables to improve health [2], spinach enrichment at 20% contributes to a ~4% increase in vegetable consumption for every slice of bread, together with a several-fold increase in the intake of micronutrients and bioactive compounds, particularly folate, calcium, iron, nitrate, nitrate and polyphenols. The intake of folate, iron and vitamin A is often deficient in some sections of the population globally, causing anaemia and other health issues [71], suggesting the new bread formulation may benefit individuals at risk of deficiency in meeting their nutritional needs. For instance, consuming an average of 76 g/day from two slices of spinach-enriched bread [72] would increase the daily values to 10–23% and 15% for iron and folate, respectively, instead of only 4–8% and 7% from white bread (where the recommended daily intake values for Australian adults are 8–18 mg and 400 µg for iron and folate, respectively [73]).

Nitrates and nitrites regulate blood pressure and lower hypertension, aiding cardiovascular health and non-communicable disease prevention [74,75,76,77], while polyphenols, a group of bioactive compounds in fruits and vegetables, offer potential health benefits. It is worth noting that certain flavonoids can inhibit carbohydrate-digesting enzymes, α-amylase and α-glucosidase, which may contribute to better glycaemic control and the management of diabetes [12,78,79]. For example, quercetagetin, initially isolated from spinach leaves [10], exhibited substantial inhibitory action against sucrase, maltase and isomaltase [12]. Ultimately, spinach-enriched bread addresses nutrient gaps in the diet and supports better health outcomes.

## 5. Conclusions

The incorporation of fresh spinach into bread significantly enhanced the nutrient composition, physical characteristics and sensory quality of bread, and at 20% *w*/*w*, it was acceptable to consumers. Spinach enrichment increased protein, fibre, iron, folate, niacin, pyridoxine, riboflavin, magnesium, potassium, zinc, calcium, vitamins A, C, and E, nitrate, nitrite and polyphenols. The improved pasting properties of spinach-enriched bread, characterised by increased viscosities and decreased pasting time and temperature, contributed to a more porous bread structure and enhanced textural properties. The interactions between spinach fibres and gluten proteins, along with gluten added to compensate for flour substitution, played a key role in these improvements. Sensory evaluation revealed that higher spinach concentrations led to decreased visual appeal, less preferred taste, odour, and overall liking, with a decrease in purchase/consumption intention. However, bread with 20% spinach was mostly comparable to white bread in terms of sensory attributes. In addition, this approach offers a sustainable solution for the valorisation of food waste, contributing to both consumer health and environmental sustainability.

## Figures and Tables

**Table 1:** Bread formulations for white bread and spinach-enriched bread.

Ingredients [g (%) (*w*/*w*)]	Control Bread	Spinach Bread (%) (*w*/*w*)			
		10%	20%	30%	40%
1. Bread flour	480 (54.0)	450 (51.6)	440 (49.5)	420 (47.3)	400 (45.0)
2. Warm water	355 (39.9)	283 (31.8)	212 (23.9)	141 (15.9)	70 (7.9)
3. Gluten substitute	0.0	2.3 (0.3)	4.6 (0.5)	6.9 (0.8)	9.2 (1.0)
4. Spinach	0.0	89.7 (10.1)	178.4 (20.1)	267.1 (30.1)	355.8 (40.0)
5. Yeast (*Saccharomyces cerevisiae*)	10 (1.1)	10 (1.1)	10 (1.1)	10 (1.1)	10 (1.1)
6. Bread improver	10 (1.1)	10 (1.1)	10 (1.1)	10 (1.1)	10 (1.1)
7. Sugar, caster	10 (1.1)	10 (1.1)	10 (1.1)	10 (1.1)	10 (1.1)
8. Butter	10 (1.1)	10 (1.1)	10 (1.1)	10 (1.1)	10 (1.1)
9. Salt	5 (0.6)	5 (0.6)	5 (0.6)	5 (0.6)	5 (0.6)
10. Gluten	8.9 (1.0)	8.9 (1.0)	8.9 (1.0)	8.9 (1.0)	8.9 (1.0)
Total weight (g)	888.9	888.9	888.9	888.9	888.9

Ingredients: 1–4 varied in concentration, while ingredients 5–10 were consistent throughout the bread formulation. Values are in weight (g) with the percentage of total weight shown in brackets.

**Table 2:** Bread yield percentage and total weight of baked loaf after spinach enrichment.

	Control White Bread	Spinach-Enriched Bread			
		10%	20%	30%	40%
Yield (%)	84 ± 1.5 ^a^	82 ± 2.1 ^ab^	81 ± 1.5 ^ab^	80 ± 1.5 ^ab^	80 ± 0.0 ^b^
Weight (g)	749 ± 15.7 ^a^	726 ± 17.0 ^ab^	723 ± 14.8 ^ab^	712 ± 12.1 ^b^	712 ± 1.5 ^b^

Calculated percentage yield. Different letters indicate significant differences at *p* < 0.05; values are presented as mean ± SD, *n* = 3.

**Table 3:** Nutritional composition of spinach and comparative macronutrient, energy and ash content in control white bread and spinach-enriched bread.

Values (/100 g Baked Bread)	Spinach/100 g	Control	10% Spinach	*p*-Value	20% Spinach	*p*-Value	30% Spinach	*p*-Value	40% Spinach	*p*-Value
Energy (kJ)	82	1053 ± 22	1061± 25	0.7132	1040 ± 21	0.4789	1029 ± 18	0.2118	1003 ± 2 *	0.0163
Protein (g)	2.40	9.04 ± 0.18	9.53 ± 0.22 *	0.0405	9.78 ± 0.19 **	0.0083	10.14 ± 0.17 **	0.0016	10.35 ± 0.02 ***	0.0002
Total fat (g)	0.30	2.26 ± 0.05	2.32 ± 0.06	0.2426	2.32 ± 0.04	0.1757	2.34 ± 0.04	0.0721	2.33 ± 0.01	0.0514
Saturated fat (g)	0.00	0.87 ± 0.02	0.89 ± 0.02	0.3046	0.89 ± 0.02	0.3465	0.90 ± 0.02	0.1926	0.89 ± 0.00	0.2378
Trans Fatty Acids (g)	0.00	0.06 ± 0.01	0.07 ± 0.00	0.1161	0.07 ± 0.00	0.1161	0.07 ± 0.00	0.1161	0.07 ± 0.00	0.1161
Polyunsaturated fat (g)	0.00	0.49 ± 0.01	0.48 ± 0.01	0.2879	0.46 ± 0.01 *	0.0390	0.45 ± 0.01 **	0.0080	0.43 ± 0.00 ***	0.0005
Monounsaturated fat (g)	0.00	0.36 ± 0.01	0.37 ± 0.01	0.2879	0.37 ± 0.01	0.3739	0.37 ± 0.01	0.3739	0.36 ± 0.00	1.0000
Carbohydrate (g)	0.60	47.17 ± 0.97	46.85 ± 1.09	0.7208	45.24 ± 0.89	0.0640	44.07 ± 0.76 *	0.0121	42.22 ± 0.09 ***	0.0009
Sugars (g)	0.60	2.49 ± 0.06	2.59 ± 0.06	0.0946	2.63 ± 0.05 *	0.0335	2.69 ± 0.05 **	0.0085	2.71 ± 0.01 **	0.0023
Starch (g)	0.00	44.68 ± 0.93	44.25 ± 1.03	0.6218	42.61 ± 0.84 *	0.0457	41.38 ± 0.71 **	0.0081	39.51 ± 0.08 ***	0.0007
Dietary fibre (g)	2.50	2.02 ± 0.05	2.33 ± 0.06 **	0.0018	2.58 ± 0.05 ****	0.0001	2.85 ± 0.05 ****	<0.0001	3.10 ± 0.01 ****	<0.0001
Ash (g)	1.60	1.15 ± 0.03	1.37 ± 0.03 ***	0.0006	1.56 ± 0.03 ****	0.0001	1.77 ± 0.03 ****	<0.0001	1.95 ± 0.01 ****	<0.0001

Spinach nutrient data/100 g, adapted from [29]. Control white bread and spinach-enriched bread nutrient profile values are presented as mean ± SD, *n* = 3. Significant differences compared with control bread are denoted by asterisks: * = *p* < 0.05, ** = *p* < 0.01, *** = *p* < 0.001, **** = *p* < 0.0001. Control white bread and spinach-enriched bread data were obtained and analysed using FoodWorks10. The retention factor is applied during the analysis using FoodWorks10 to account for cooking and processing nutrient loss.

**Table 4:** Nutritional composition of spinach and comparative micronutrient and bioactive compound content in control white bread and spinach-enriched bread.

Values (/100 g Baked Bread)	Spinach/100 g	Control	10% Spinach	*p*-Value	20% Spinach	*p*-Value	30% Spinach	*p*-Value	40% Spinach	*p*-Value
Thiamin (mg)	0.06	0.28 ± 0.01	0.28 ± 0.01	0.6433	0.28 ± 0.01	1.0000	0.28 ± 0.01	1.0000	0.27 ± 0.00	0.1161
Riboflavin (mg)	0.16	0.00 ± 0.00	0.02 ± 0.00	-	0.04 ± 0.00	-	0.06 ± 0.00	-	0.08 ± 0.00	-
Niacin (mg)	0.40	0.96 ± 0.02	1.02 ± 0.03 *	0.0474	1.04 ± 0.02 **	0.0093	1.08 ± 0.02 **	0.0021	1.10 ± 0.01 ***	0.0004
Niacin eq. (mg)	0.99	2.90 ± 0.06	3.05 ± 0.07 *	0.0435	3.13 ± 0.06 **	0.0094	3.24 ± 0.06 **	0.0020	3.31 ± 0.01 ***	0.0003
Pyridoxine (mg)	0.16	0.05 ± 0.01	0.06 ± 0.01 *	0.0241	0.08 ± 0.00 ***	0.0006	0.10 ± 0.00 ****	0.0001	0.12 ± 0.00 ****	<0.0001
Folic acid (µg)	0.00	38.59 ± 0.80	38.14 ± 0.89	0.5443	36.64 ± 0.73 *	0.0353	35.5 ± 0.61 **	0.0059	33.79 ± 0.07 ***	0.0005
Food folate (µg)	110.0	36.3 ± 0.8	47.4 ± 1.1 ****	0.0001	57.5 ± 1.1 ****	<0.0001	68.4 ± 1.2 ****	<0.0001	78.4 ± 0.2 ****	<0.0001
Total vitamin A eq. (µg)	469.0	11.5 ± 0.2	50.3 ± 1.2 ****	<0.0001	88.8 ± 1.8 ****	<0.0001	128.9 ± 2.2 ****	<0.0001	167.6 ± 0.3 ****	<0.0001
Retinol (µg)	0.00	11.11 ± 0.23	11.46 ± 0.27	0.1643	11.51 ± 0.23	0.0995	11.68 ± 0.20 *	0.0332	11.68 ± 0.03 *	0.0137
β carotene (µg)	1920.0	2.04 ± 0.04	227.5 ± 5.3 ****	<0.0001	452.3 ± 8.9 ****	<0.0001	686.1 ± 11.8 ****	<0.0001	913.0 ± 1.8 ****	<0.0001
Vitamin C (mg)	27.00	0.00 ± 0.00	2.34 ± 0.06 ****	<0.0001	4.67 ± 0.09 ****	<0.0001	7.09 ± 0.12 ****	<0.0001	9.44 ± 0.02 ****	<0.0001
Vitamin E (mg)	1.30	0.09 ± 0.00	0.24 ± 0.01 ****	<0.0001	0.39 ± 0.01 ****	<0.0001	0.55 ± 0.01 ****	<0.0001	0.70 ± 0.00 ****	<0.0001
Tocopherol, α (mg)	1.30	0.09 ± 0.00	0.24 ± 0.01 ****	<0.0001	0.39 ± 0.01 ****	<0.0001	0.55 ± 0.01 ****	<0.0001	0.70 ± 0.00 ****	<0.0001
Iron (mg)	3.20	0.88 ± 0.02	1.28 ± 0.03 ****	<0.0001	1.67 ± 0.03 ****	<0.0001	2.07 ± 0.04 ****	<0.0001	2.45 ± 0.01 ****	<0.0001
Zinc (mg)	0.60	0.79 ± 0.02	0.87 ± 0.02 **	0.0080	0.92 ± 0.02 **	0.0013	0.99 ± 0.02 ***	0.0003	1.05 ± 0.01 ****	<0.0001
Calcium (mg)	53.0	16.80 ± 0.34	23.55 ± 0.55 ****	0.0001	29.83 ± 0.58 ****	<0.0001	36.54 ± 0.62 ****	<0.0001	42.80 ± 0.08 ****	<0.0001
Potassium (mg)	570.0	130.3 ± 2.7	200.4 ± 4.7 ****	<0.0001	266.9 ± 5.3 ****	<0.0001	337.4 ± 5.8 ****	<0.0001	403.8 ± 0.8 ****	<0.0001
Magnesium (mg)	68.0	25.15 ± 0.52	33.3 ± 0.77 ****	0.0001	40.80 ± 0.80 ****	<0.0001	48.85 ± 0.84 ****	<0.0001	56.27 ± 0.12 ****	<0.0001
Phosphorus (mg)	46.0	98.1 ± 2.0	104.1 ± 2.4 *	0.0303	107.5 ± 2.1 **	0.0053	112.0 ± 1.9 ***	0.0010	114.9 ± 0.2 ****	0.0001
Sodium (mg)	21.0	267.0 ± 5.6	280.7 ± 6.5	0.0963	284.3 ± 5.6 *	0.0352	290.8 ± 5.0 **	0.0085	293.0 ± 0.6 **	0.0021
Selenium (µg)	0.00	8.21 ± 0.17	8.26 ± 0.20	0.7376	8.09 ± 0.16	0.4310	8.00 ± 0.14	0.1720	7.80 ± 0.02 *	0.0130
Iodine (µg)	0.00	5.69 ± 0.12	5.59 ± 0.13	0.3786	5.34 ± 0.10 *	0.0186	5.14 ± 0.09 **	0.0030	4.86 ± 0.01 ***	0.0003
Linoleic (g)	-	0.45 ± 0.01	0.44 ± 0.01	0.4918	0.43 ± 0.01 *	0.0249	0.42 ± 0.01 **	0.0075	0.40 ± 0.00 ***	0.0010
α-Linolenic (g)	-	0.04 ± 0.00	0.04 ± 0.00	-	0.04 ± 0.01	0.3739	0.04 ± 0.01	0.3739	0.03 ± 0.00	-
Nitrate (mg)	296.0	0.00 ± 0.00	36.6 ± 0.9 ****	<0.0001	73.1 ± 1.4 ****	<0.0001	111.1 ± 1.9 ****	<0.0001	147.9 ± 0.3 ****	<0.0001
Nitrite (mg)	3.80	0.00 ± 0.00	0.46 ± 0.01 ****	<0.0001	0.92 ± 0.02 ****	<0.0001	1.41 ± 0.03 ****	<0.0001	1.87 ± 0.01 ****	<0.0001
Kaempferol (mg)	7.86	0.00 ± 0.00	0.97 ± 0.02 ****	<0.0001	1.94 ± 0.04 ****	<0.0001	2.95 ± 0.05 ****	<0.0001	3.93 ± 0.01 ****	<0.0001
Quercetin (mg)	5.87	0.00 ± 0.00	0.73 ± 0.02 ****	<0.0001	1.45 ± 0.03 ****	<0.0001	2.20 ± 0.04 ****	<0.0001	2.93 ± 0.01 ****	<0.0001
Luteolin (mg)	1.11	0.00 ± 0.00	0.14 ± 0.01 ****	<0.0001	0.28 ± 0.01 ****	<0.0001	0.42 ± 0.01 ****	<0.0001	0.55 ± 0.01 ****	<0.0001
Total polyphenols	248.1	0.00 ± 0.00	30.0 ± 1.0 ****	<0.0001	60.6 ± 1.5 ****	<0.0001	92.7 ± 1.5 ****	<0.0001	123.3 ± 0.6 ****	<0.0001

Spinach nutrient data per 100 g, adapted from [29], except total vitamin E eq. adapted from [30], nitrate and nitrite information adapted from [17], kaempferol, quercetin, luteolin and total polyphenol content was adapted from [11]. Control white bread and spinach-enriched bread nutrient profile values are presented as mean ± SD, *n* = 3. Significant differences compared with control bread are denoted by asterisks: * = *p* < 0.05, ** = *p* < 0.01, *** = *p* < 0.001, **** = *p* < 0.0001. Control white bread and spinach-enriched bread data were obtained and analysed using FoodWorks10, where values for nitrate, nitrite and polyphenols in raw spinach were manually entered into the software. “Niacin eq.” accounts for niacin and potential niacin that can be synthesised from the tryptophan content in the food. “Total vitamin A eq.” represents the sum of preformed (retinol) and provitamin A carotenoids. Eq.: equivalent. The retention factor is applied during the analysis using FoodWorks10 to account for cooking and processing nutrient losses.

**Table 5:** Pasting properties of wheat flour starch enriched with freeze-dried spinach powder.

Primary Test	Freeze-Dried Spinach Concentration Added to the Dough				
	Control	10%	20%	30%	40%
Peak viscosity (cP)	1619 ^c^	2395 ^c^	2972 ^bc^	3920 ^b^	7539 ^a^
Trough viscosity (cP)	893 ^c^	1133 ^bc^	1216 ^bc^	1742 ^b^	3237 ^a^
Breakdown viscosity (cP)	727 ^d^	1262 ^cd^	1757 ^bc^	2179 ^b^	4302 ^a^
Final viscosity (cP)	1923 ^b^	2018 ^b^	2015 ^b^	2720 ^b^	4961 ^a^
Setback viscosity (cP)	1030 ^b^	885 ^b^	799 ^b^	978 ^b^	1724 ^a^
Peak time (min)	5.7 ^ab^	5.8 ^a^	5.8 ^a^	5.6 ^b^	4.9 ^c^
Pasting temperature (°C)	85.9 ^a^	65.9 ^b^	65.2 ^b^	65.6 ^b^	63.3 ^c^

Different letters indicate significant differences at *p* < 0.05. Values are the mean of duplicate samples.

**Table 6:** Panellist comments and recommendations during sensory analysis.

Spinach Bread	Comments and Recommendations
0% (286)	“I love this product” “a bit dry” “a bit bitter and bland, so more salt”
10% (492)	“a bit crumbly” “good balance of taste of bread and spinach” “the smell is still quite strong” “the product is a bit dry, lacks moisture” “feels stale” “bitter and bland, it also tastes eggy” “overall flavour needs improving, maybe more salt” “reduce dryness”
20% (219)	“similar to 492 but slightly more sour” “less spinach taste” “the smell of the bread needs to be improved” “very distinct flavour” “add salt” “bitter and bland”
30% (537)	“still a slight taste of spinach that overpowers the bread” “strong aftertaste” “the smell is quite strong, decrease it” “it is bitter” “very bitter taste”
40% (153)	“a little too fluffy” “strong leafy taste, mouldy odour” “this one has an unpleasant aftertaste” “masking the smell of the spinach” “less spinach taste” “the herbal smell of the bread is quite strong, it might need to be improved, (make it less)” “it is bitter so check if spoilt and if not add more salt to enhance taste” “it is bitter” “very strong taste of spirulina, taste like grass. The texture of the bread is lovely, but need to mask the flavour” “too earthy” “make it less bitter”

Note: Samples were presented as randomised and blinded 3-digit codes.

## Data Availability

The original contributions presented in the study are included in the article/Supplementary Material; further inquiries can be directed to the corresponding author.

## Supplementary Materials

The following supporting information can be downloaded at https://www.mdpi.com/article/10.3390/foods13152401/s1, Video S1: Timelapse of dough fermentation for control and dough enriched with 20% and 40% spinach. Research Data S1: Raw data supporting reported results.

## References

[1] Sarker A., Ahmmed R., Ahsan S.M., Rana J., Ghosh M.K., Nandi R. (2024). A Comprehensive Review of Food Waste Valorization for the Sustainable Management of Global Food Waste. Sustain. Food Technol..

[2] World Health Organisation Increasing Fruit and Vegetable Consumption to Reduce the Risk of Noncommunicable Diseases. https://www.who.int/tools/elena/interventions/fruit-vegetables-ncds#:~:text=WHO%20Recommendations,the%20risk%20of%20certain%20NCDs.

[3] Petersen K.S., Kris-Etherton P.M. (2021). Diet Quality Assessment and the Relationship between Diet Quality and Cardiovascular Disease Risk. Nutrients.

[4] Shang X., Liu J., Zhu Z., Zhang X., Huang Y., Liu S., Wang W., Zhang X., Tang S., Hu Y. (2023). Healthy Dietary Patterns and the Risk of Individual Chronic Diseases in Community-Dwelling Adults. Nat. Commun..

[5] Sharifi-Rad J., Rodrigues C.F., Sharopov F., Docea A.O., Can Karaca A., Sharifi-Rad M., Kahveci Karıncaoglu D., Gülseren G., Şenol E., Demircan E. (2020). Diet, Lifestyle and Cardiovascular Diseases: Linking Pathophysiology to Cardioprotective Effects of Natural Bioactive Compounds. Int. J. Environ. Res. Public Health.

[6] Cicero A.F.G., Fogacci F., Stoian A.P., Vrablik M., Al Rasadi K., Banach M., Toth P.P., Rizzo M. (2021). Nutraceuticals in the Management of Dyslipidemia: Which, When, and for Whom? Could Nutraceuticals Help Low-Risk Individuals with Non-Optimal Lipid Levels?. Curr. Atheroscler. Rep..

[7] Iqbal I., Wilairatana P., Saqib F., Nasir B., Wahid M., Latif M.F., Iqbal A., Naz R., Mubarak M.S. (2023). Plant Polyphenols and Their Potential Benefits on Cardiovascular Health: A Review. Molecules.

[8] Roughani A., Miri S.M. Spinach: An Important Green Leafy Vegetable and Medicinal Herb. Proceedings of the 2nd International Conference on Medicinal Plants, Organic Farming, Natural and Pharmaceutical Ingredients.

[9] Sri Lasya C., Author Chokkara Sri Lasya C. (2022). Spinach and Its Health Benefits: A Review. Pharma Innov. J..

[10] Zane A., Wender S.H. (1961). Flavonols in Spinach Leaves. J. Org. Chem..

[11] Phenol-Explorer Food Composition-Spinach, Raw. http://phenol-explorer.eu/contents/food/272#folin-assay.

[12] Barber E., Houghton M.J., Williamson G. (2021). Flavonoids as Human Intestinal α-Glucosidase Inhibitors. Foods.

[13] Liu R.H. (2013). Health-Promoting Components of Fruits and Vegetables in the Diet. Adv. Nutr..

[14] Frankowska A., Jeswani H.K., Azapagic A. (2019). Environmental Impacts of Vegetables Consumption in the UK. Sci. Total Environ..

[15] Statista Bread—Worldwide. https://www.statista.com/outlook/cmo/food/bread-cereal-products/bread/worldwide.

[16] Willett W., Manson J., Liu S. (2002). Glycemic Index, Glycemic Load, and Risk of Type 2 Diabetes. Am. J. Clin. Nutr..

[17] Food Standards Australia New Zealand Survey of Nitrates and Nitrites in Food and Beverages in Australia. https://www.foodstandards.gov.au/science-data/surveillance/surveyofnitrates.

[18] Junejo S.A., Rashid A., Yang L., Xu Y., Kraithong S., Zhou Y. (2021). Effects of Spinach Powder on the Physicochemical and Antioxidant Properties of Durum Wheat Bread. LWT.

[19] American Association of Cereal Chemists (1988). Approved Methods of the AACC–Method 74-09.

[20] Baker A.E., Walker C.E., Kemp K. (1988). An Optimum Compression Depth for Measuring Bread Crumb Firmness. Cereal Chemistry.

[21] Chou S., Meng X., Cui H., Zhang S., Wang H., Li B. (2019). Rheological and Pasting Properties of Maize, Wheat and Rice Starch as Affected by Apple Polyphenols. Int. J. Food Prop..

[22] Han X., Zhang M., Zhang R., Huang L., Jia X., Huang F., Liu L. (2020). Physicochemical Interactions between Rice Starch and Different Polyphenols and Structural Characterization of Their Complexes. LWT.

[23] (2017). Sensory Analysis—Methodology—General Guidance 2017.

[24] Mammasse N., Schlich P. (2014). Adequate Number of Consumers in a Liking Test. Insights from Resampling in Seven Studies. Food Qual. Prefer..

[25] Nyitrai Á., Urbin Á., Nagy B.V., Sipos L. (2022). Novel Approach in Sensory Color Masking: Effects of Colored Environments on Chocolates with Different Cocoa Content. Food Qual. Prefer..

[26] Singh-Ackbarali D., Maharaj R. (2014). Sensory Evaluation as a Tool in Determining Acceptability of Innovative Products Developed by Undergraduate Students in Food Science and Technology at The University of Trinidad and Tobago. J. Curric. Teach..

[27] Food Standards Australia and New Zealand (FSANZ) Food Composition Databases. https://www.foodstandards.gov.au/science-data/food-composition-databases.

[28] Xyris FoodWorks Introduction to Dietary Analysis with FoodWorks 10. https://support.xyris.com.au/hc/en-us/article_attachments/360002775975.

[29] Food Standards Australia and New Zealand Australian Food Composition Database—Release 2.0. https://afcd.foodstandards.gov.au/fooddetails.aspx?PFKID=F008761.

[30] U.S. Department of Agriculture Spinach, Raw. https://fdc.nal.usda.gov/fdc-app.html#/food-details/168462/nutrients.

[31] Kumar R., Khatkar B.S. (2017). Thermal, Pasting and Morphological Properties of Starch Granules of Wheat (*Triticum aestivum* L.) Varieties. J. Food Sci. Technol..

[32] Mohd Shukri A., Cheng L.-H. (2023). The Properties of Different Starches under the Influence of Glucono-Delta-Lactone at Different Concentrations. Foods.

[33] Yildiz Ö., Yurt B., Baştürk A., Toker Ö.S., Yilmaz M.T., Karaman S., Dağlıoğlu O. (2013). Pasting Properties, Texture Profile and Stress–Relaxation Behavior of Wheat Starch/Dietary Fiber Systems. Food Res. Int..

[34] Ragaee S., Abdel-Aal E.-S.M. (2006). Pasting Properties of Starch and Protein in Selected Cereals and Quality of Their Food Products. Food Chem..

[35] Kaushal P., Kumar V., Sharma H.K. (2012). Comparative Study of Physicochemical, Functional, Antinutritional and Pasting Properties of Taro (*Colocasia esculenta*), Rice (*Oryza sativa*) Flour, Pigeonpea (*Cajanus cajan*) Flour and Their Blends. LWT-Food Sci. Technol..

[36] Pacheco A.F.C., Pacheco F.C., dos Santos F.R., Cunha J.S., Paiva P.H.C., Banger S.P. (2024). Starch Paste Properties. Methods and Protocols in Food Science.

[37] Fagundes G.A., Rocha M., Salas-Mellado M.M. (2018). Improvement of Protein Content and Effect on Technological Properties of Wheat Bread with the Addition by Cobia (*Rachycentron canadum*). Food Res..

[38] Sari K.I., Rafisa A. (2023). Chewing and Swallowing Patterns for Different Food Textures in Healthy Subjects. Int. J. Dent..

[39] Guiné R.P.F., Roque A.R.F., Seiça F.F.A., Batista C.E.O. (2016). Effect of Chemical Pretreatments on the Physical Properties of Kiwi. ETP Int. J. Food Eng..

[40] Paredes J., Cortizo-Lacalle D., Imaz A.M., Aldazabal J., Vila M. (2022). Application of Texture Analysis Methods for the Characterization of Cultured Meat. Sci. Rep..

[41] Trịnh K.T., Glasgow S. On the Texture Profile Analysis Test. Proceedings of the Chemeca 2012.

[42] Szczesniak A.S. (2002). Texture Is a Sensory Property. Food Qual. Prefer..

[43] Kidmose U., Edelenbos M., Christensen L.P., Hegelund E. (2005). Chromatographic Determination of Changes in Pigments in Spinach (*Spinacia oleracea* L.) During Processing. J. Chromatogr. Sci..

[44] Waseem M., Akhtar S., Manzoor M.F., Mirani A.A., Ali Z., Ismail T., Ahmad N., Karrar E. (2021). Nutritional Characterization and Food Value Addition Properties of Dehydrated Spinach Powder. Food Sci. Nutr..

[45] Sengev A.I., Abu J.O., Gernah D.I. (2013). Effect of *Moringa oleifera* Leaf Powder Supplementation on Some Quality Characteristics of Wheat Bread. Food Nutr. Sci..

[46] Okakpu K.G., Offia-Olua B.I., Okakpu C.J., Okpara C.M. (2023). Quality Characteristics of Bread Made from Flour Blends of Wheat, Cooking Banana and Mungbean. J. Adv. Food Sci. Technol..

[47] Begum R., Chowdhury M.A.F., Hasan M.R., Rahman M.F., Rahman M.H., Alim M.A. (2023). Efficacy of Freeze-Dried Carrot Pomace Powder in Improving the Quality of Wheat Bread. Food Res..

[48] Ranawana V., Campbell F., Bestwick C., Nicol P., Milne L., Duthie G., Raikos V. (2016). Breads Fortified with Freeze-Dried Vegetables: Quality and Nutritional Attributes. Part II: Breads Not Containing Oil as an Ingredient. Foods.

[49] Wang J., Rosell C.M., Benedito de Barber C. (2002). Effect of the Addition of Different Fibres on Wheat Dough Performance and Bread Quality. Food Chem..

[50] Ficco D.B.M., Muccilli S., Padalino L., Giannone V., Lecce L., Giovanniello V., Del Nobile M.A., De Vita P., Spina A. (2018). Durum Wheat Breads “high in Fibre” and with Reduced in Vitro Glycaemic Response Obtained by Partial Semolina Replacement with Minor Cereals and Pulses. J. Food Sci. Technol..

[51] Wang Y., Pan Y., Zhou C., Li W., Wang K. (2024). Effects of Kiwifruit Dietary Fibers on Pasting Properties and In Vitro Starch Digestibility of Wheat Starch. Nutrients.

[52] Zhu F. (2015). Interactions between Starch and Phenolic Compound. Trends Food Sci. Technol..

[53] Cappelli A., Oliva N., Cini E. (2020). A Systematic Review of Gluten-Free Dough and Bread: Dough Rheology, Bread Characteristics, and Improvement Strategies. Appl. Sci..

[54] Zhou Y., Dhital S., Zhao C., Ye F., Chen J., Zhao G. (2021). Dietary Fiber-Gluten Protein Interaction in Wheat Flour Dough: Analysis, Consequences and Proposed Mechanisms. Food Hydrocoll..

[55] Gómez M., Ronda F., Blanco C.A., Caballero P.A., Apesteguía A. (2003). Effect of Dietary Fibre on Dough Rheology and Bread Quality. Eur. Food Res. Technol..

[56] Amoah I., Cairncross C., Rush E. (2021). Swallowing and Liking of Vegetable-Enriched Bread Compared With Commercial Breads as Evaluated by Older Adults. Front. Nutr..

[57] Zhou Q., Liang W., Wan J., Wang M. (2023). Spinach (*Spinacia oleracea*) Microgreen Prevents the Formation of Advanced Glycation End Products in Model Systems and Breads. Curr. Res. Food Sci..

[58] Nisha P., Singhal R.S., Pandit A.B. (2004). A Study on the Degradation Kinetics of Visual Green Colour in Spinach (Spinacea Oleracea L.) and the Effect of Salt Therein. J. Food Eng..

[59] Mohtarami F., Esmaiili M., Nouraddini M., Ostad I. (2022). Fortification of Simit Bread with Spinach Powder: Evaluation of Physico-Chemical, Textural, and Sensorial Properties. Food Sci. Technol..

[60] Khan M.A., Mahesh C., Semwal A.D., Sharma G.K. (2015). Effect of Spinach Powder on Physico-Chemical, Rheological, Nutritional and Sensory Characteristics of Chapati Premixes. J. Food Sci. Technol..

[61] Kiranawati T.M., Hidayati L., Saputri A.M.J., Sari R.A., Susanto H. (2021). The Analysis of Bread Quality from Moringa Oleifera (Kelor) Leaf Flour. Proceedings of the International Conference on Life Sciences and Technology.

[62] Osakabe N., Shimizu T., Fujii Y., Fushimi T., Calabrese V. (2024). Sensory Nutrition and Bitterness and Astringency of Polyphenols. Biomolecules.

[63] Kebede B.T., Grauwet T., Tabilo-Munizaga G., Palmers S., Vervoort L., Hendrickx M., Van Loey A. (2013). Headspace Components That Discriminate between Thermal and High Pressure High Temperature Treated Green Vegetables: Identification and Linkage to Possible Process-Induced Chemical Changes. Food Chem..

[64] Masanetz C., Guth H., Grosch W. (1998). Fishy and Hay-like off-Flavours of Dry Spinach. Z. Leb. Und-Forsch. A.

[65] Díaz-Mula H.M., Marín A., Jordán M.J., Gil M.I. (2017). Off-Odor Compounds Responsible for Quality Loss of Minimally Processed Baby Spinach Stored under MA of Low O_2_ and High CO_2_ Using GC–MS and Olfactometry Techniques. Postharvest Biol. Technol..

[66] Mehta V., Desai N., Perwez A., Nemade D., Dawoodi S., Zaman S. (2017). Bin ACE Alzheimer’s: The Role of Vitamin A, C and E (ACE) in Oxidative Stress Induced Alzheimer’s Disease. J. Med. Res. Innov..

[67] Purić M., Rabrenović B., Rac V., Pezo L., Tomašević I., Demin M. (2020). Application of Defatted Apple Seed Cakes as a By-Product for the Enrichment of Wheat Bread. LWT.

[68] Czarnowska-Kujawska M., Starowicz M., Barišić V., Kujawski W. (2022). Health-Promoting Nutrients and Potential Bioaccessibility of Breads Enriched with Fresh Kale and Spinach. Foods.

[69] Galla N.R., Pamidighantam P.R., Karakala B., Gurusiddaiah M.R., Akula S. (2017). Nutritional, Textural and Sensory Quality of Biscuits Supplemented with Spinach (*Spinacia oleracea* L.). Int. J. Gastron. Food Sci..

[70] Lee H.W., Bi X., Henry C.J. (2023). Assessment of Oxalates and Phytic Acid Contents in Asian Green Leafy Vegetables: Dietary Recommendations. Food Humanit..

[71] World Health Organisation (2023). Accelerating Anaemia Reduction: A Comprehensive Framework for Action.

[72] Grafenauer S., Curtain F. (2018). An Audit of Australian Bread with a Focus on Loaf Breads and Whole Grain. Nutrients.

[73] National Health and Medical Research Council (2006). Nutrient Reference Values for Australia and New Zealand Executive Summary.

[74] Kapil V., Khambata R.S., Robertson A., Caulfield M.J., Ahluwalia A. (2015). Dietary Nitrate Provides Sustained Blood Pressure Lowering in Hypertensive Patients. Hypertension.

[75] Bondonno C.P., Blekkenhorst L.C., Liu A.H., Bondonno N.P., Ward N.C., Croft K.D., Hodgson J.M. (2018). Vegetable-Derived Bioactive Nitrate and Cardiovascular Health. Mol. Aspects Med..

[76] Bondonno C.P., Yang X., Croft K.D., Considine M.J., Ward N.C., Rich L., Puddey I.B., Swinny E., Mubarak A., Hodgson J.M. (2012). Flavonoid-Rich Apples and Nitrate-Rich Spinach Augment Nitric Oxide Status and Improve Endothelial Function in Healthy Men and Women: A Randomized Controlled Trial. Free Radic. Biol. Med..

[77] Jonvik K.L., Nyakayiru J., Pinckaers P.J., Senden J.M., van Loon L.J., Verdijk L.B. (2016). Nitrate-Rich Vegetables Increase Plasma Nitrate and Nitrite Concentrations and Lower Blood Pressure in Healthy Adults. J. Nutr..

[78] Zhu J., Chen C., Zhang B., Huang Q. (2020). The Inhibitory Effects of Flavonoids on α-Amylase and α-Glucosidase. Crit. Rev. Food Sci. Nutr..

[79] Tadera K., Minami Y., Takamatsu K., Matsuoka T. (2006). Inhibition of α-Glucosidase and α-Amylase by Flavonoids. J. Nutr. Sci. Vitaminol..

